# Effects of Dancing on Cognition in Healthy Older Adults: a Systematic Review

**DOI:** 10.1007/s41465-018-0103-2

**Published:** 2018-10-18

**Authors:** David Predovan, Anne Julien, Alida Esmail, Louis Bherer

**Affiliations:** 1 Centre de Recherche, Institut Universitaire de Gériatrie de Montréal, 4565 Chemin Queen-Mary, Montreal, Québec H3W 1W5 Canada; 2 Département de Psychologie, Université du Québec à Montréal, Montreal, QC Canada; 3 Centre de Recherche, Institut de Cardiologie de Montréal, Montreal, QC Canada; 4 PERFORM Centre and Department of Psychology, Concordia University, Montreal, QC Canada; 5 École de réadaptation, Faculté de Médecine, Université de Montréal, Montreal, QC Canada; 6 Département de Médecine, Université de Montréal, Montreal, QC Canada

**Keywords:** Aging, Dancing, Cognition, Prevention

## Abstract

A growing body of research emphasizes the benefits of physical activity and exercise over the lifespan and especially in elderly populations. However, few studies have evaluated the impact of dance as a physical activity or exercise on cognition in healthy older adults. This review investigated if dance could be used as a promising alternative intervention to address physical inactivity and to cognitively stimulate older adults. This systematic review reports the effects of dancing in a healthy older adult population based on intervention studies using the EMBASE, Web of Science, and Ovid Medline databases. The Cochrane collaboration’s tool for assessing risk of bias was used to assess each article quality. Seven out of 99 articles met the inclusion criteria, representing a total of 429 older adults (70% women), with a mean age of 73.17 years old. Dance interventions, lasting between 10 weeks and 18 months, were related to either the maintenance or improvement of cognitive performance. This systematic review suggests that dance as an intervention in the elderly could help improve or maintain cognition. This review outlines some of the possible mechanisms by which dance could positively impact cognition in older adults, addresses shortcomings in the existing literature, and proposes future research avenues.

## Background

As life expectancy increases, nearly two billion people in the world will be aged 60 years and over in 2050 (Bloom 2011). Therefore, a better understanding of interventions that may delay the onset or progression of age-related diseases that affect cognition is paramount. In this regard, a growing body of research has emphasized the benefits of physical activity over the lifespan and notably, during later ages (Bherer et al. 2013). Physical activity is generally defined as any activity involving bodily movements that results in energy expenditure (Caspersen et al. 1985). As such, a meta-analysis of prospective longitudinal studies (Sofi et al. 2011) has shown evidence that being physically active can lessen the risk of cognitive decline. Intervention studies also support the benefits of exercise training on cognition in sedentary older adults (Berryman et al. 2014). On that account, it seems that physical activity fits closely into the framework of delaying cognitive decline due to secondary aging. However, motivation for engaging in physical activity is often lacking in older adults and should be among the top priorities for research in this field. One way to motivate individuals to engage in physical activity and increase their adherence is to expand the range of physical activities available to them. However, studies documenting the impact of various types of exercise, such as soft gym and dancing (Dhami et al. 2014), are still scarce. Dancing is a particularly promising type of intervention that could potentially stimulate both physical and cognitive functions in older adults. Involving multisensory stimulation, social interaction, and the learning of sequences of movements, the act of dancing could lead to synergic effects. In order to examine the latest evidence on the subject, the present review investigated whether dance could have an impact on cognition in older adults.

According to a recent report on the physical activity patterns of different age groups, dancing is observed as being one of the most popular physical activities in American women aged 45 years and older (Fan et al. 2013). Dancing as a type of physical activity is defined as “a patterned, rhythmic movement in space and time” (Murrock and Graor 2014). Studying the act of dancing could shed light on many aspects of our everyday behavior such as motor control (e.g., balance and posture), timing and synchronization of movements, learning sequences of movements, and understanding how we memorize, perceive, and imagine them. A review of the literature (Keogh et al. 2009) highlights a number of benefits associated with different types of dance, such as significant improvement in aerobic power, muscular endurance, strength, flexibility of lower limbs, posture, balance, and gait.

As of now, few studies have attempted to quantify the effects of dancing on cognition in a healthy elderly population. A prospective study (Verghese et al. 2003) tracked a cohort of 469 older adults, aged between 75 and 85 years old, for a median time of 5 years and observed that the practice of dance as a leisure activity, several days or more per week, was associated with a reduced risk of developing dementia. Further evidence for this comes from a cross-sectional study (Kattenstroth et al. 2010), where the effect of multiyear amateur dance activities (average of 16.5 years, 1.33 ± 0.24 h per week) in a healthy elderly population (mean age = 72 years) was examined by comparing a group of dancers (*n* = 24) to an age- and education-matched control group of non-dancers (*n* = 38). A significant difference was observed to the advantage of dancers on a subset of the 30 items taken from the Raven Standard Progressive Matrices (RSPM), a measure of fluid intelligence. However, no significant differences were reported on a non-verbal geriatric concentration test (AKT). According to the authors, the difference in performance between groups could be due to the lack of individuals having poor performance on these tests in the dance group, contrary to the control group. Thereby, they concluded that participation in a dance group may help preserve cognition from further decline but might not improve cognition itself. Furthermore, absence of a correlation between the total number of years of dance experience, the average time spent dancing each week, and the cognitive performance could indicate that the benefit of dancing may reach a plateau after a certain point of time or simply that other predispositions specific to the individuals of the dance group (Kreutz 2008) explain these results. In another cross-sectional study (Verghese 2006), older social dancers (*n* = 24, mean age = 80 years) matched (age, gender, education, levels of physical and cognitive leisure activities) to a group of older non-dancers (*n* = 84) were compared on episodic memory tasks (Free and Cued Selective Reminding test) and other cognitive tests (Digit Span test, Digit Symbol test, Block Design test, Verbal fluency test and Trail making tests). No advantage was found for the dancers over the non-dancers. Limitations addressed by the authors included the “dose” of dancing in the social dancer group that was not considered and the small sample size. Lastly, as both groups were matched on the levels of participation in leisure activities other than dancing, it is possible that, overall, similar levels of lifestyle activities could preclude the association between dancing and cognitive gain.

To ensure a better quantification of the dose-response relationship of dancing on physical and cognitive well-being and to provide a better understanding of the underlying mechanisms, systematic evaluation of a formal dance program offers an appropriate experimental design. As of now, a limited number of intervention studies have assessed if dancing carried out as a structured program could be beneficial for the cognition of older adults. The present systematic review aimed to examine the current state of the literature based on this evidence. Furthermore, highlights of some of the limits and methodological weaknesses of the present literature are presented and avenues for future research will be discussed.

## Methods

### Data Sources and Search Criteria

A systematic search of the literature with the EMBASE, Web of Science, and Ovid Medline databases was conducted independently by two of the authors (D.P., A.J.). Articles were selected using the following keywords “Dancing” OR “Dance” combined with “Aging” OR “Older Adults” combined with “Cognition” OR “Memory” OR “Executive functions.” All relevant articles in English (including protocol descriptions, pilot studies, reviews), published between 2000 and May 2018, were selected. This selection was also supplemented by a hand search of the bibliographical references of relevant articles.

### Study Selection and Quality Assessment

To be included in the final selection, articles had to meet the following criteria: (a) participants had to be healthy sedentary older adults (older than 60 years old) who were not expert dancers (i.e., had no formal dancing training), (b) the intervention study design had to include one group performing dance (that was not combined with another intervention) and an active or passive (i.e., waiting list) control group, and (c) the outcome included assessment of neuropsychological functions or report of cognitive performance. Thereafter, intervention studies were rated using the Cochrane collaboration’s tool for assessing risk of bias (Higgins et al. 2011) using five of the six original bias, as the blinding of participants and personnel to the intervention is often difficult, if at all possible in the context of an exercise intervention.

## Results

### Results of the Search Strategy

Based on the Preferred Reporting Items for Systematic Reviews and Meta-Analyses (PRISMA) Statement (Moher et al. 2009), Fig. 1 presents the four-phase flow diagram which leads to the final selection of articles. A database search and handsearching identified 99 articles. Thirty-four of the 99 articles were removed based on the criteria of manuscript types. The remaining articles were screened for relevant content and eligibility. This phase lead to the exclusion of 58 articles due to the following criteria (population = 16, intervention = 14, assessment = 28). Finally, seven studies were included in this review. A summary of the selected studies is presented in Table 1.

**Fig. 1:**
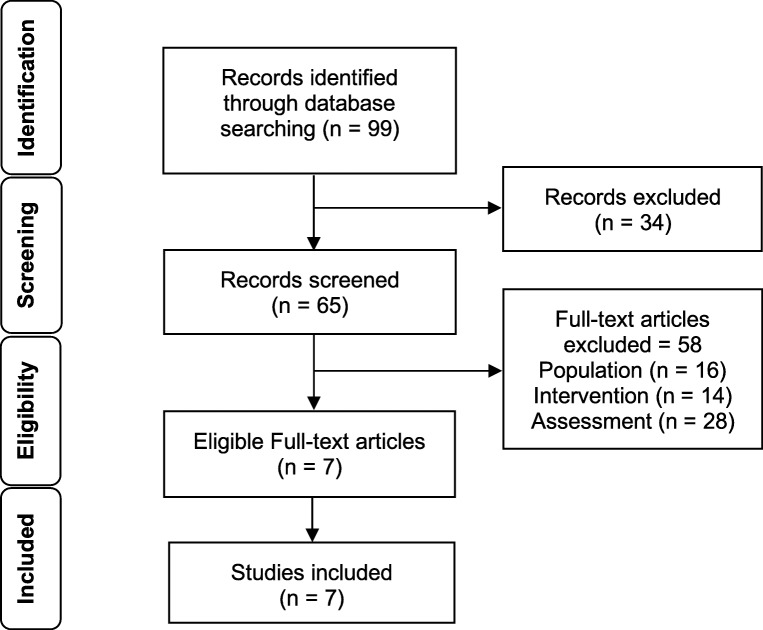
PRISMA flow diagram summarizing the results of the search strategy

**Table 1:** Characteristics of the studies included

Reference	Dance intervention	Control	Duration	Assessment: cognition
Coubard et al. (2011)	Contemporary dance (*n* = 16)	Fall prevention (*n* = 67) Tai Chi Chuan (*n* = 27)	60 min/week for approx. 5.7 months	Arithmetic word problems, Stroop test, Rule shift cards test*
Hackney et al. (2015)	Adapted tango (*n* = 62)	Health education (*n* = 12)	20 × 90 min for 4 months	Montreal Cognitive Assessment, Reverse Corsi Blocks, Brooks Spatial Task, Trail-Making Test Part B, Body Position Spatial Task*
Hamacher et al. (2015)	Dancing (*n* = 23)	Health-related exercise (*n* = 22)	2 × 90 min/week for 6 months	Motor-cognitive dual task*: walking while reciting successive subtraction
Kattenstroth et al. (2013)	Dancing (Agilando™) (*n* = 25)	No intervention (*n* = 10)	60 min/week for 6 months	Non-verbal geriatric concentration test (AKT), Frankfurt Attention Inventory*, Repeatable Battery of Neuropsychological Status*, Non-Verbal Learning test, Raven Standard Progressive Matrices*
Kosmat and Vranic 2016	Dancing (*n* = 12)	Non-dance active group (*n* = 12)	45 min/week for 10 weeks	Modified Auditory Verbal Learning test (AVLT)*, Wisconsin Card Sorting Test (WCST)*
Merom et al. (2016)	Ballroom dancing (*n* = 60)	Home walking (*n* = 55)	2 × 60 min/week for 8 months	Trail Making tests, Stroop Color-Word test, Digit Span Backwards test, Rey Auditory Verbal Learning test: immediate and delayed verbal recall, BVMT Visuospatial recall*
Mueller et al. (2017)	Dance (*n* = 26)	Sport (*n* = 26)	2 × 90 min/week for 6 months, then 1 × 90 min/week for 12 months	Modified Rey Auditory Verbal Learning test*, Test of Attentional Performance (TAP)*

*Significant (*P* < 0.05) post-intervention performance improvements for the dance intervention

### Methodological Quality

**Fig. 2:**
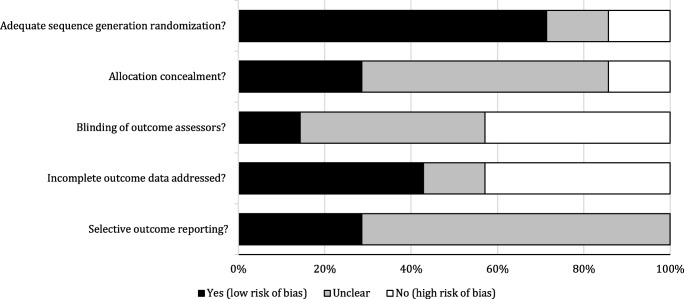
Risk-of-bias graph

Using the Cochrane collaboration’s tool for assessing risk of bias, D.P. and A.J. independently assessed the risk of bias for all the studies included. Figure 2 shows the risk of bias. Five of the seven articles were evaluated as high quality on the random sequence generation criterion. High-quality mention was also given to two articles on the allocation concealment criterion, one article on the blinding of outcome assessment criterion, three articles on the incomplete outcome data criterion, and two articles on the selective reporting criterion. Only the study of Merom et al. (2016) was rated high quality on each criterion.

### Participants and Study Characteristics

A combined total of 429 older adults (70% women), with a mean age of 73.17 years old, were recruited in the seven selected studies. A follow-up assessment was done in two of the studies at 3 months (Hackney et al. 2015) and 5 months (Kosmat and Vranic 2016) after their respective interventions. Recruitment was done primarily in Europe (Coubard et al. 2011; Hamacher et al. 2015; Kattenstroth et al. 2013; Kosmat and Vranic 2016; Mueller et al. 2017) and to a lesser degree in America (Hackney et al. 2015) and Oceania (Merom et al. 2016).

### Characteristics of the Interventions

With a duration of 10 weeks, a study (Kosmat and Vranic 2016), examined the impact of dancing sessions compared to a non-dancing activity session which each lasted 45 min per week. The dancing sessions started with warm-up exercises (15 min), and the core dance component (30 min) was divided between learning choreography (motor sequences of increasing complexity; for 20 min) and learning to Slow Waltz (10 min). Another study, who had a 4-month intervention (Hackney et al. 2015), compared an adapted tango group to a health education group. The program encompassed 20 sessions of 90 min each. The dance session was divided between a 20-min standing warm-up and 40-min tango program. In another study (Coubard et al. 2011), a contemporary dance group, a fall prevention group, and a Tai Chi Chuan group participated in a weekly 60-min session during their 5.7-month-long program. The dance session was divided into four phases: opening (5-min), warm-up (20-min), improvisation (30-min), and closure/cool-down (5-min). Among the selected studies, two had a duration of 6 months. In one of them, a dancing group was compared to a health-related exercise group (Hamacher et al. 2015). Each group participated in two 90-min (15-min warm-up, 60 min of activity and 15-min cool-down) sessions weekly. The health-related exercise included three activities: endurance training, strength-endurance training, and flexibility training, lasting 20 min each. The dance group had to learn specific dancing skills such as line dance, jazz dance, rock and roll, Latin-American dance, and square dance. The other 6-month study (Kattenstroth et al. 2013) examined the impact of a specific type of dance Agilando ™ to a control group. The dance sessions lasted 60 min and were performed weekly alone or in a group. A typical dance session was divided into a 20-min warm up and a 40-min phase where participants had to learn sequences of movement. With an intervention duration of 8 months, one study (Merom et al. 2016) examined the impact of ballroom dancing against a home walking activity. Participants in each group had to participate in two 60-min sessions weekly. Different types of dance were learned such as ballroom dances, Rock and Roll, Foxtrot, Waltz, and some Latin (Salsa and Rumba). Lastly, with a duration of 18 months (Mueller et al. 2017), the described dance program included different types of dance such as line dance, jazz dance, rock “n” roll, and square dance. The complexity of the choreographies increased over time, as well as the beats per minute in the music.

### Adherence

Adherence was computed for four of the seven studies. For a program duration of 4 months, 6 months and 8 months, the adherence was respectively 71% (dance) vs 83% (control) (Hackney et al. 2015), 83% (dance) vs 73% (control) (Hamacher et al. 2015), and 67% (dance) vs 71% (control) (Merom et al. 2016). In another study (Mueller et al. 2017), adherence at month 6 was 77% (dance) and 69% (sport group) and at month 18 was 46% (dance) and 38% (sport group).

### Cognition Measurements

General cognition was mainly assessed using the Mini-Mental State Examination (MMSE) (Folstein et al. 1975) and on one occasion with the Telephone Interview of Cognitive Status (TICS) (Welsh et al. 1993) and the Montreal Cognitive Assessment (MoCA) (Nasreddine et al. 2005). A multitude of cognitive domains was assessed such as executive functions (Wisconsin Card Sorting Test (WCST), Stroop Color-Word test, Rule shift cards test, Trail-Making test), short-term auditory-verbal memory (Rey Auditory Verbal Learning test, Digit Span Backwards test), and visuo-spatial short-term memory (Reverse Corsi Blocks, Brooks Spatial task, Brief Visuospatial Memory test (BVMT)). Diverse measurements of attention, such as the Arithmetic word problems test, the Frankfurt Attention Inventory, a non-verbal geriatric concentration test (AKT), and the Test of Attentional Performance (TAP), were also used. A battery to assess overall cognitive decline (Repeatable Battery of Neuropsychological Status (RBANS)), a Non-Verbal Learning test and a test assessing fluid intelligence (Raven Standard Progressive Matrices), were also used. Lastly, complex tasks involving motor-cognitive function (Body Position Spatial task) or motor-cognitive dual task (walking while reciting successive subtraction) was implemented in two studies. Post-intervention performance improvements were observed on a cognitive flexibility task (Coubard et al. 2011), a motor-cognitive function task (Hackney et al. 2015), a motor-cognitive dual-task (Hamacher et al. 2015), the Frankfurt Attention Inventory test, the RBANS, a Non-Verbal Learning test (Kattenstroth et al. 2013), verbal short-term memory, verbal long-term memory and attention (Mueller et al. 2017), a modified Auditory Verbal Learning test, the WCST (Kosmat and Vranic 2016), and the BVMT (Merom et al. 2016).

## Discussion

Overall, some evidence suggests that dance interventions could be an efficient way to improve or maintain cognition in older adults. The effect of dancing on cognition depends most likely on a multitude of mechanisms (Foster 2013). One component is the complexity of coordination learning found in the act of dancing. Evidence suggests that exercise programs promoting coordination could have different effects on the brain, compared to aerobic exercise training designed to improve cardiorespiratory fitness (Voelcker-Rehage and Niemann 2013). This effect can be emphasized by the study of the acute effects of two aerobic dance exercise workouts in an older adult population (40 min at approximately 40% heart rate reserve) on a switching task (Kimura and Hozumi 2012). Older adults (*n* = 34) were either assigned randomly to a freestyle workout consisting of learning several patterns of movement or a combination-style workout consisting of similar patterns that had to be joined together. Remarkably, the task-switching reaction time was reduced significantly only in the combination-style workout group.

Another mechanism by which a dance program could affect cognition is via its effect on depression symptoms. Independently from learning a movement sequence, the effect of socialization and music, an essential part of dancing, can evoke emotional experiences and physiological reactions (Murrock and Higgins 2009) such as arousal, which could in turn affect mood. Furthermore, when seen as a participatory art, the expressive and communicative parts of dancing could affect the mood of the dancer (Noice et al. 2014). Lastly, in an older adults group, it has been shown that the presence of music during physical exercise training could improve cognitive performance (Satoh et al. 2014).

## Future Directions

More studies are needed to further validate the hypothesis that dancing could simultaneously improve cognition and physical capacity. In order to reduce the number of shortcomings found in the present literature, future studies should have an active control group. This would reduce the following confounding factors: effect of the social interaction and effect of displacement to attend the training sessions. Other methodological improvements could be (1) the implementation of measurements at several time points to understand the evolution of changes, (2) the use of extensive neuropsychological testing and of computerized cognitive tasks, (3) the consideration of vascular risk factors and other chronic diseases known to impact cognition, (4) an examination of potential gender effects, (5) the comparison of adherence between different types of dance to reduce the attrition bias, (6) the inclusion of depression and anxiety measurements, (7) an exhaustive description of the program content to improve the reproducibility of these studies, (8) the implementation of a randomized controlled trial design to reduce the selection bias and improved baseline comparability, (9) the examination of the relationship between improvement in gait performance and cognition, (10) the comparison of different types of dance programs (i.e., different levels of complexity and improvisation, aerobic intensity, or interaction (alone, with a partner or in a group)), and (11) the use of neuroimaging techniques to further develop our comprehension of the underlying brain mechanisms by which dance and/or dance-related activities can help improve or maintain cognitive performance in older adults. Several imaging studies examined “the neural basis of human dance” (Brown et al. 2006). Some studies have investigated the visual and motor imagery involved in dancing (Cross et al. 2013); however, imaging techniques have rarely been used in examining the effects of a dance intervention program (Burzynska et al. 2017; Rehfeld et al. 2017); therefore, knowledge on the anatomical and functional brain changes associated with the effects of a dance program in the older adults is limited.

## Conclusion

This review examined the most recent evidence regarding the impact of dancing on cognitive performance in older adults. Some evidence suggests that dance as an intervention for older adults could improve and help maintain cognition. This review positions itself in the context of successful aging (Rowe and Kahn 1997) to promote the development of strategies and interventions for older adults so that they can continue to function at a high level and contribute to society. The hope is that this review will bring attention towards innovative interventions, such as dancing, to increase the range of activities available for the older adults that can be carried out alone, in groups or with a partner.
